# Antibiotic Resistance Mechanisms in Bacteria: Relationships Between Resistance Determinants of Antibiotic Producers, Environmental Bacteria, and Clinical Pathogens

**DOI:** 10.3389/fmicb.2018.02928

**Published:** 2018-11-30

**Authors:** Elizabeth Peterson, Parjit Kaur

**Affiliations:** Department of Biology, Georgia State University, Atlanta, GA, United States

**Keywords:** self-resistance mechanisms, antibiotic resistance, *Streptomyces*, clinical pathogens, horizontal gene transfer, resistance gene dissemination, environmental bacteria, producer bacteria

## Abstract

Emergence of antibiotic resistant pathogenic bacteria poses a serious public health challenge worldwide. However, antibiotic resistance genes are not confined to the clinic; instead they are widely prevalent in different bacterial populations in the environment. Therefore, to understand development of antibiotic resistance in pathogens, we need to consider important reservoirs of resistance genes, which may include determinants that confer self-resistance in antibiotic producing soil bacteria and genes encoding intrinsic resistance mechanisms present in all or most non-producer environmental bacteria. While the presence of resistance determinants in soil and environmental bacteria does not pose a threat to human health, their mobilization to new hosts and their expression under different contexts, for example their transfer to plasmids and integrons in pathogenic bacteria, can translate into a problem of huge proportions, as discussed in this review. Selective pressure brought about by human activities further results in enrichment of such determinants in bacterial populations. Thus, there is an urgent need to understand distribution of resistance determinants in bacterial populations, elucidate resistance mechanisms, and determine environmental factors that promote their dissemination. This comprehensive review describes the major known self-resistance mechanisms found in producer soil bacteria of the genus *Streptomyces* and explores the relationships between resistance determinants found in producer soil bacteria, non-producer environmental bacteria, and clinical isolates. Specific examples highlighting potential pathways by which pathogenic clinical isolates might acquire these resistance determinants from soil and environmental bacteria are also discussed. Overall, this article provides a conceptual framework for understanding the complexity of the problem of emergence of antibiotic resistance in the clinic. Availability of such knowledge will allow researchers to build models for dissemination of resistance genes and for developing interventions to prevent recruitment of additional or novel genes into pathogens.

## Introduction: A Brief Historical Perspective

Selman Waksman, a prominent researcher in the field of actinomycetes in the early part of the twentieth century, described the term antibiotic as a chemical compound generated from microorganisms that inhibits or destroys other microbes (Hopwood, 2007; Davies and Davies, 2010). Most antibiotics in use today originated from the phylum *Actinobacteria* with nearly 80% of actinobacterial-derived antibiotics produced by soil-dwelling bacteria of the genus *Streptomyces* (Barka et al., 2016). Before the discovery of natural antibiotics, synthetic compounds, including salvarsan, sulfa drugs and quinolones, were in use as chemotherapeutic agents (Aminov, 2010). Penicillin was the first natural antibiotic to be discovered accidentally by Alexander Fleming in 1928 when the *Penicillium* fungus contaminated a culture plate in his laboratory, however, penicillin was not developed for use until the late 1930s (Hopwood, 2007). Penicillin inhibits cell wall synthesis and was found to be very effective against Gram-positive but not against Gram-negative bacteria (due to the presence of the outer membrane) or the tubercle bacillus (because of the extra thick cell wall) (Hopwood, 2007). Following the discovery of penicillin by Fleming, other scientists, including Rene Dubos and Selman Waksman, started a deliberate search for antibacterial agents among soil microorganisms, including bacteria and fungi. It was soon realized that antibacterial activity was most often present in actinomycete cultures and less often in other bacteria or fungi. During this period, several antibiotics were discovered in the screens designed by these scientists but many of these were of little use in the clinic due to their toxicity in animals. The next biggest discovery came about in 1943, resulting in identification of streptomycin produced by *Streptomyces griseus*. Streptomycin inhibits protein synthesis by binding to the 30S subunit of the prokaryotic ribosome and was found to be effective not only against Gram-negative bacteria but also against the tubercle bacillus (Hopwood, 2007). With the discovery of streptomycin, the golden age of antibiotic discovery and development (1940–1990) ensued. This involved efforts of many academic institutions and major pharmaceutical companies in the United States and other countries. Currently, antibiotics affecting almost every process in the bacterial cell are known. Based on their structure and mode of action, at least seven major groups of antibiotics have been described. These include β-lactams (inhibit cell wall synthesis), aminoglycosides (protein synthesis), macrolides (protein synthesis), tetracyclines (protein synthesis), daptomycin (cell membrane function), platensimycin (fatty acid biosynthesis), and glycopeptides (cell wall synthesis).

It is only natural that organisms which produce antibiotics should also contain self-resistance mechanisms against their own antibiotics. In addition, co-existence of producer and non-producer bacteria is also believed to have resulted in co-evolution of resistance mechanisms in non-producing environmental bacteria. Resistance determinants found in these two groups of bacteria have garnered significant attention in recent years because of their possible link with the emergence of resistance in pathogenic clinical isolates (Surette and Wright, 2017; Martinez, 2018). Indeed, with the global epidemic of antibiotic resistance unfolding before us, it is important to understand the origin of these determinants in pathogens. This review article provides an up-to-date understanding of the antibiotic self-resistance mechanisms found in producer soil bacteria of the genus *Streptomyces* and explores relationships between resistance determinants found in producer and non-producer soil and environmental bacteria and the clinical pathogenic bacteria. The topic of self-resistance in producer bacteria has never before been reviewed in its entirety, while antibiotic resistance mechanisms in clinical isolates have been extensively described (Munita and Arias, 2016). Therefore, resistance mechanisms of clinical isolates are not discussed in detail in this article. Critical additional information about clinical isolates is, however, provided in a separate section following description of self-resistance in *Streptomyces*. These two sections were kept separate in this review because resistance mechanisms of producers and clinical isolates are currently at very different levels of understanding. In the last sections of this review, origins of resistance determinants in clinical strains and potential mechanisms for their mobilization are discussed. Although every attempt has been made to be inclusive of all available literature, the information on each topic addressed in this review is broad and constantly growing, therefore any omission is unintentional. Where possible, references to additional literature and review articles are provided for further reading.

## Self-Resistance Mechanisms in Producer Organisms

**FIGURE 1:**
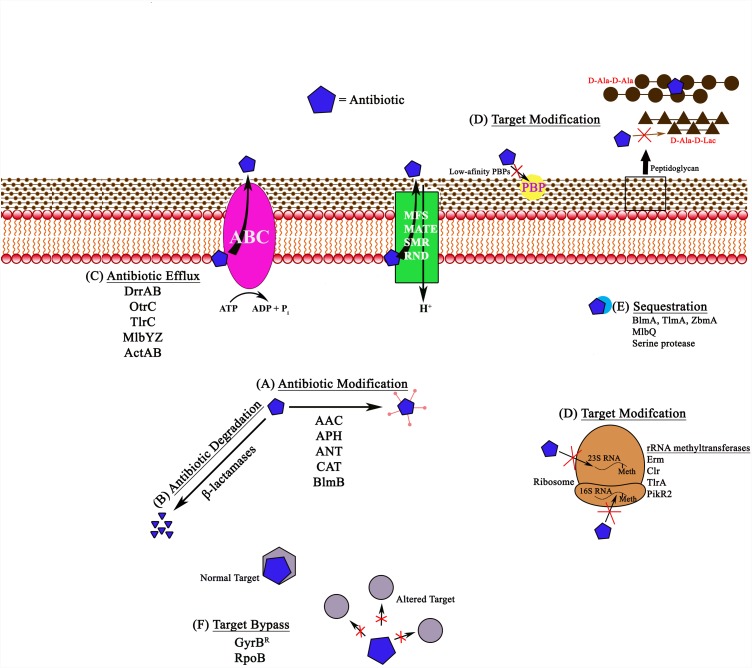
Schematic representation of different antibiotic resistance mechanisms in bacteria, shown with examples. **(A)** Antibiotic modification involves the addition of acetyl, phosphate, or adenyl groups to aminoglycosides by *N*-acetyl transferases (AAC), *O*-phosphotransferases (APH), and *O*-adenyltransferases (ANT). Other examples include chloramphenicol acetyl transferases (CAT) and bleomycin *N*-acetyltransferases (BlmB). **(B)** Antibiotic degradation is observed with β-lactamases, which hydrolyze the antibiotic. **(C)** Antibiotic efflux pumps remove the antibiotic from the cell using energy from ATP hydrolysis in ABC pumps like DrrAB, OtrC, TlrC, and MlbYZ, or proton gradients in MFS, MATE, SMR, and RND family pumps. **(D)** Target modification includes various target alterations, such as 23S rRNA or 16S rRNA methylation, alterations in the peptidoglycan precursors (for example, in the case of glycopeptides), or synthesis of alternate low-affinity targets (PBPs) that reduce or completely block antibiotic (penicillins) from associating with the target. **(E)** Antibiotic sequestration involves proteins that can associate with the antibiotic and block them from reaching their targets. **(F)** Target bypass involves generation of additional antibiotic targets or subunits that are not susceptible to binding of the antibiotic. Meth, methylation.

Antibiotic producing bacteria contain a variety of sophisticated mechanisms for self-defense against their own antibiotics (Figure 1 and Table 1). Very often they contain multiple mechanisms simultaneously to ensure complete protection from the biologically active molecules produced by them. Interestingly, the genetic determinants for self-resistance are almost always clustered together with the antibiotic biosynthesis genes, and their expression is co-regulated (Mak et al., 2014). The following section highlights major biochemical categories of self-defense mechanisms found in producer organisms with specific examples provided for each category.

**Table 1:** Antibiotic self-resistance mechanisms in producer bacteria.

Mechanism of antibiotic resistance	Selected examples	Gene location	Reference
Antibiotic modification/degradation	Aminoglycoside modifying enzymes (AME):AAC; APH; ANT Streptomycin-6-phosphotransferase	Chromosome *S. griseus* (*smk)*	Shinkawa et al., 1985; Mak et al., 2014
	β-lactamases Class A,B,C	Chromosome *Streptomyces* species	Ogawara, 2016b
Antibiotic efflux	ABC transporter DrrAB (Dox) OtrC (oxytetracycline)	Chromosome *S. peucetius (drr*AB*) S. rimosus (otr*C*)*	Yu et al., 2012; Li et al., 2014
	MFS transporter OtrB (oxytetracycline) Mfs1 (natamycin)	Chromosome *S. rimosus* (*otr*B) *S. chattanoogensis* (*mfs1*)	Ohnuki et al., 1985; Reynes et al., 1988; Wang et al., 2017
Antibiotic sequestration by special proteins	Sequestration TlmA, BlmA, ZbmA (bleomycin)	Chromosome *S. hindustanus* (*tlm*A); *S. verticillus (blm*A*); S. flavoviridis (zbm*A*)*	Gatignol et al., 1988; Sugiyama et al., 1994; Rudolf et al., 2015
Antibiotic target modification	Low affinity penicillin-binding proteins (PBP) Class A Class B	Chromosome *Streptomyces* species	Ogawara, 2015, 2016a
	Peptidoglycan remodeling (Glycopeptides) VanH_st_, DdlM, VanX_st_ VanH_aov_, DdlN, VanX_aov_	Chromosome *S. toyocaensis* (*van*H_st,_ *ddl*M, *van*X_st_); *A. orientalis* (*van*H_aov_, *ddl*N, *van*X_aov_)	Marshall et al., 1998; Binda et al., 2014
	23S rRNA methylation (MLS) Clr, PikR1, PikR2	Chromosome*S. caelestis* (*clr*) *S. venezuelae* (*pik*R1, *pik*R2)	Calcutt and Cundliffe, 1990; Almutairi et al., 2015
	16S rRNA methylation (Aminoglycosides) PCT, Sgm methylase	Chromosome *S. pactum* (*pct*) *M. zionesis* (*sgm*)	Ballesta and Cundliffe, 1991; Kojic et al., 1992
Antibiotic target bypass	DNA gyrase subunit B (novobiocin)	Chromosome *S. sphaeroides (gyrB^R^)*	Schmutz et al., 2003
Antibiotic target protection	Antibiotic removal DrrC (Dox) OtrA (oxytetracycline)	Chromosome *S. peucetius* (*drr*C) *S. rimosus* (*otr*A)	Doyle et al., 1991; Mak et al., 2014; Prija and Prasad, 2017


**AAC*, N-acetyl transferases; APH, O-phosphotransferases; ANT, O-adenyltransferases; ABC, ATP-binding cassette superfamily; MFS, major facilitator superfamily; Dox, doxorubicin; MLS, macrolides, lincosamides, and streptogramins; PCT, pactamycin methylase; Sgm, sisomicin-gentamicin resistance methylase.*

### Antibiotic Modification or Degradation

Antibiotic modification is a commonly used strategy for rendering an antibiotic ineffective, especially in the case of aminoglycoside antibiotics (for example, kanamycin, gentamycin, and streptomycin), chloramphenicol, and β-lactams. A large number of aminoglycoside modification enzymes (AMEs), including *N*-acetyl transferases (AAC), *O*-phosphotransferases (APH), and *O*-adenyltransferases (ANT) that acetylate, phosphorylate, or adenylylate the aminoglycoside antibiotic, respectively, are known to exist in producer bacteria. Although these enzymes were first identified in the producer *Streptomyces* species in the early 1970s, and they perform identical biochemical reactions to those seen in antibiotic resistant clinical strains (Walker and Walker, 1970; Benveniste and Davies, 1973), a direct correlation between synthesis of aminoglycosides and the presence of modification enzymes in producer *Streptomyces* is not always evident. For example, some species may not produce antibiotics but still contain modification enzymes, and vice versa. One exception is streptomycin resistance, where a direct correlation between antibiotic synthesis and the role of modification enzymes in self-resistance has indeed been established. Streptomycin resistance in the producer *S. griseus* involves the function of the modification enzyme streptomycin 6-phosphotransferase that converts streptomycin to an inactive precursor streptomycin-6-phosphate. Streptomycin 6-phosphotransferase is the last enzyme in the biosynthetic pathway, and the expression of the gene encoding this enzyme is co-regulated with biosynthesis genes (Shinkawa et al., 1985; Mak et al., 2014).

Other than the example of streptomycin, the biological function of AMEs in the producer organisms has been a subject of unresolved debate for a long time. It has been speculated that these enzymes may not be directly involved in resistance in producers, but instead may perform other metabolic functions (Benveniste and Davies, 1973; Martinez, 2018). This claim is supported by comparative sequence analyses showing that the AMEs are quite diverse and are encoded by a large group of unrelated genes, thus suggesting that they might have originated by multiple convergent paths resulting in a similar function (Shaw et al., 1993). Other studies have also pointed out potential structural and sequence similarities between AMEs of producers and cellular metabolic enzymes, including similarity between APH and protein kinases and between AAC and protein acylases (Heinzel et al., 1988; Piepersberg et al., 1988; Davies and Wright, 1997), implying that the modification enzymes might have been co-opted from housekeeping metabolic enzymes for antibiotic resistance. Thus many unanswered questions remain, which deserve a careful and systematic investigation. Future investigations should also determine if most aminoglycoside biosynthesis gene clusters found in producer *Streptomyces* contain genes for modification enzymes and whether these enzymes play a role in self-resistance.

Modification of the antibiotic as a mechanism for self-defense is also seen for other classes of antibiotics. For example, the bleomycin (BLM) family members [bleomycin (BLM), tallysomycin (TLM), phleomycin (PLM) and zorbamycin (ZBM)] are subject to acetylation. BLMs and TLMs are produced by *Streptomyces verticillus* and *Streptoalloteichus hindustanus*, respectively, and their biosynthesis gene clusters contain genes for *N*-acetyltransferases, BlmB and TlmB. These enzymes carry out acetylation of the metal-free forms of BLMs and TLMs, thus preventing correct formation of the metal-binding domain of these antibiotics (Coughlin et al., 2014). Finally, chloramphenicol is another antibiotic that can be acetylated by a large and widely distributed group of enzymes known as chloramphenicol acetyl transferases (CATs). Although these enzymes have been shown to be very prevalent in clinical strains (Schwarz et al., 2004) and are also likely to be common in *Streptomyces*, only a few reports of identification of CAT enzymes from *Streptomyces* species are available (Murray et al., 1989).

In contrast to the modification of antibiotics described above, resistance to β-lactam antibiotics is normally conferred by antibiotic-hydrolyzing enzymes known as β-lactamases. These enzymes are widespread among *Streptomyces*, and, together with similar enzymes found in pathogenic and non-pathogenic bacteria, they constitute the ‘β-lactamase superfamily’ of proteins (Sattler et al., 2015; Ogawara, 2016b). β-lactamases are generally grouped into four classes (A,B,C,D) based on their amino acid sequence and use of a catalytic serine or zinc ion (King et al., 2016). In a recent phylogenetic screen conducted by Ogawara, it was found that diverse β-lactamases belonging to classes A, B, and C exist in many *Streptomyces* species. However, a clear relationship between the level of β-lactamases and the degree of resistance to β-lactam antibiotics in these species has not been established (Ogawara, 2016b). This is due to the fact that most *Streptomyces* species produce β-lactamases constitutively, and their production is not related to resistance or synthesis of β-lactams. As discussed previously for AMEs, *Streptomyces* β-lactamases also exhibit diverse species-specific properties, again suggesting convergent evolution from different proteins to perform the same function, i.e., hydrolysis of the β-lactam ring (Allen et al., 2009). The presence of β-lactamases in producers also presents an evolutionary conundrum – how can β-lactams and β-lactamases co-exist simultaneously in producer cells? Perhaps these enzymes play alternative cellular functions in *Streptomyces*, are expressed at low levels, or are expressed in a growth phase different from biosynthesis? Overall, therefore, it has been proposed that β-lactamases may not play an important role in resistance in *Streptomyces* species, which may instead involve the function of low-affinity penicillin binding proteins (PBPs) (Ogawara, 2015) discussed in Section “Target Modification/Bypass/Protection Mechanisms” in this article.

### Antibiotic Efflux

Efflux of antibiotics is another commonly used mechanism for self-resistance, although it usually occurs in conjunction with other mechanisms, such as modification of the antibiotic or the target. The best studied example of antibiotic efflux among producers is found in *Streptomyces peucetius*, which produces two closely related anticancer antibiotics, daunorubicin (Dnr) and doxorubicin (Dox). These two antibiotics intercalate with DNA preventing further rounds of replication. Efflux of these antibiotics in *S. peucetius* occurs by an ABC (ATP Binding Cassette) family transporter DrrAB coded by the *drr*AB genes embedded within the gene cluster responsible for biosynthesis of these antibiotics (Guilfoile and Hutchinson, 1991). The DrrAB system has been studied in significant molecular and biochemical detail. The DrrAB pump is assembled from two subunits each of the ABC protein DrrA and the integral membrane protein DrrB. DrrA protein functions as the catalytic nucleotide binding domain (NBD). DrrB protein functions as the carrier protein and forms the transmembrane domain (TMD). In an *in vitro* assay using inverted membrane vesicles, the DrrAB proteins were shown to carry out efflux of Dox in ATP or GTP-dependent manner (Li et al., 2014). Because of the location of the *drr*AB genes in the Dox biosynthesis gene cluster, this system is considered to be a dedicated transporter of Dnr and Dox in *S. peucetius*. Interestingly, however, recent studies showed that DrrAB pump is a multidrug transporter with broad substrate specificity, and it can transport many previously known MDR (multidrug resistance) pump substrates such as ethidium bromide, Hoechst 33342, verapamil, and vinblastine, among others (Li et al., 2014). In this regard, the DrrAB system is similar to the mammalian ABC multidrug transporter *P*-glycoprotein (Pgp), which is overexpressed in human cancer cells and is one of the major causes for failure of chemotherapy (Chufan et al., 2015). Recent studies showed that critical aromatic residues, contributed by multiple helices in DrrB, form part of a large (common) drug-binding pocket (Li et al., 2014; Brown et al., 2017). Mammalian Pgp also uses aromatic residues to provide flexibility in substrate recognition, suggesting a common origin for these proteins and an aromatic residue-based mechanism for polyspecificity that is conserved over large evolutionary distances (Chufan et al., 2015; Szewczyk et al., 2015).

Interestingly, OtrC found in oxytetracycline producer *Streptomyces rimosus* is another example of a self-resistance efflux system that exhibits multidrug specificity. Self-resistance in *S. rimosus* is conferred by two efflux proteins: OtrB (previously known as TetB) located in the biosynthesis cluster, and OtrC located outside of the cluster (Mak et al., 2014). OtrB belongs to the major facilitator superfamily (MFS) of transport proteins, but not much is known about its mechanism of action or substrate specificity (Ohnuki et al., 1985; Reynes et al., 1988; Mak et al., 2014). OtrC protein is an ABC family protein, and like DrrAB, it also confers resistance to multiple antibiotics and MDR substrates, including ampicillin, oxytetracycline, doxorubicin, ethidium bromide, ofloxacin and vancomycin (Yu et al., 2012; Mak et al., 2014). Interestingly, the DrrAB and OtrC systems are quite homologous and show high sequence conservation in the previously identified motifs, including the DEAD and the LDEVLF motifs of DrrA (Zhang et al., 2010, 2015) and the EAA-like motif in DrrB (Kaur et al., 2005; Yu et al., 2012), suggesting close evolutionary links between efflux systems of different producer organisms.

It might be expected that efflux systems found in producer organisms would be specific for the antibiotic that the system is dedicated for. Surprisingly, however, the two examples (DrrAB and OtrC) discussed above suggest polyspecific drug recognition in these systems. This raises interesting questions. Why is a multidrug transporter needed in a producer organism? What is the origin of DrrAB-like polyspecific antibiotic and drug efflux systems? Are most efflux systems associated with biosynthetic gene clusters polyspecific? Did these systems evolve from possibly even more ancient broad-spectrum efflux systems that might have served as general defense mechanisms against toxins in environmental bacteria? That transporters involved in antibiotic resistance could have been repurposed from the general defense efflux systems has been suggested previously (Dantas and Sommer, 2012; Martinez, 2018). Such an origin could explain why these systems are multi-specific, and how they could be easily adapted by different producer organisms to transport individual antibiotics synthesized by them. Analysis of many additional efflux systems found in biosynthesis clusters of producer organisms is needed to begin to formulate clear answers to these questions.

Many other examples of ABC as well as MFS transporters used for conferring self-resistance in producer organisms to lantibiotic NAI-107, polyene macrolide natamycin, tylosin, or actinorhodin are known (Rosteck et al., 1991; Xu et al., 2012; Mak et al., 2014; Pozzi et al., 2016; Wang et al., 2017). However, their molecular mechanisms and substrate specificities have not yet been elucidated.

### Antibiotic Sequestration

Sequestration involves the function of drug-binding proteins, which prevent the antibiotic from reaching its target. In producers of the bleomycin family of antibiotics, the primary mechanism of resistance involves sequestration of the metal-bound or the metal-free antibiotic (Sugiyama and Kumagai, 2002) by binding proteins TlmA, BlmA, and ZbmA in *S. hindustanus* ATCC 31158 (Gatignol et al., 1988), *S. verticillus* (Sugiyama et al., 1994, 1995), and *Streptomyces flavoviridis*, respectively (Rudolf et al., 2015). Each bleomycin-family producer member has one or more genes related to ABC transporters in their biosynthesis clusters (Du et al., 2000; Tao et al., 2007; Galm et al., 2009), which may be used to remove the antibiotics bound to binding proteins. For additional examples, see references (Sheldon et al., 1997, 1999; Pozzi et al., 2016).

### Target Modification/Bypass/Protection Mechanisms

Target modification acts as a self-resistance mechanism against several classes of antibiotics, including β-lactams, glycopeptides, macrolides, lincosamides, and streptogramins (MLS), and aminoglycosides. The β-lactam antibiotic has a similar structure to PBP substrates (peptidoglycan precursors), thus allowing the antibiotic to associate and cause acylation of the active site serine resulting in its inhibition (Yeats et al., 2002). The producer *Streptomyces* species, despite being Gram-positive, are highly resistant to penicillins, which is due to either overproduction of PBPs or synthesis of low-affinity PBPs (Ogawara, 2015). Three classes of PBPs (A, B, and C) are found in bacteria (Ogawara, 2015). Analysis of the biosynthesis clusters of β-lactam producing bacteria showed that they often contain genes for PBPs, suggesting their role in self-resistance (Liras and Martin, 2006; Ogawara, 2015). Interestingly, *Streptomyces* species contain on average more than 10 PBPs, including both Classes A and B, a number much greater than found in other *Actinobacteria*. Some of these PBPs indeed have low affinity for β-lactams most likely due to the absence of a serine/threonine protein kinase domain (STPK) (renamed PASTA) that binds β-lactams (Ogawara and Horikawa, 1980; Nakazawa et al., 1981; Coque et al., 1993; Paradkar et al., 1996; Yeats et al., 2002; Ishida et al., 2006; Ogawara, 2016a).

Glycopeptides, such as vancomycin and teicoplanin, inhibit cell wall transpeptidation and transglycosylation by associating with peptidoglycan precursors (D-Ala-D-Ala) (Binda et al., 2014). Antibiotic resistance results from a change in the peptidoglycan precursor from D-Ala-D-Ala to D-Ala-D-Lac or D-Ala-D-Ser, which has a 1000- and 6-fold reduction in affinity for the glycopeptides, respectively (Bugg et al., 1991; Billot-Klein et al., 1994). Genes conferring vancomycin resistance were initially identified in clinical strains, with the *van*A cluster (*van*HAX) on the transposon Tn*1546* being the most commonly seen. Some systems also use VanY, a D,D-carboxypeptidase to produce tetrapeptides incapable of glycopeptide binding (Binda et al., 2014). Related core *van*HAX clusters have been found in producer organisms, suggesting an evolutionary relatedness of resistance within producers and pathogens (Marshall et al., 1997, 1998). The examples include similar *van*H (Marshall et al., 1998), *van*A (Marshall and Wright, 1997, 1998), and *van*X (Lessard et al., 1998) sequences in the glycopeptide producers *Streptomyces toyocaensis* NRRL 15009 and *Amycolatopsis orientalis*. Variants on the core cluster are also reported (Schaberle et al., 2011; Binda et al., 2012; Marcone et al., 2014; Frasch et al., 2015). Other glycopeptide producers, without an obvious *van*HAX cluster, may have currently unidentified or poorly understood *van* resistance genes, such as *van*J*/sta*P (Hong et al., 2004; Novotna et al., 2012) and *van*K (Hong et al., 2005).

Target modification is also seen for MLS antibiotics, which bind to the 50S ribosomal subunit. This mechanism involves methylation of 23S rRNA at residue A-2058 by 23S rRNA methyltransferases (Douthwaite et al., 2004). Monomethylation (MLS type I) typically provides moderate level of resistance, while dimethylation (MLS type II) provides strong resistance (Fyfe et al., 2016). For further information on MLS resistance mechanisms, see reviews (Matsuoka and Sasaki, 2004; Mast and Wohlleben, 2014; Spizek and Rezanka, 2017). Finally, resistance against aminoglycosides by target modification uses 16S rRNA methyltransferases, which methylate at residue A1408 or G1405 (Shakil et al., 2008). This mechanism for self-resistance may work in conjunction with the AMEs, which were described earlier.

Other resistance mechanisms bypass the original target by producing additional low affinity targets. Examples include synthesis of additional B subunit of DNA gyrase for novobiocin resistance, alternate resistant RNA polymerase for rifamycin resistance, or an alternate fatty acid synthase for resistance to platensimycin (Blanco et al., 1984; Thiara and Cundliffe, 1988, 1989; Schmutz et al., 2003; Sanchez-Hidalgo et al., 2010; Peterson et al., 2014). Antibiotic removal from the target site provides another protective resistance mechanism. In *S. peucetius*, DrrC removes intercalated daunorubicin/doxorubicin from DNA resulting in normal transcription and replication (Prija and Prasad, 2017). In *S. rimosus*, the antibiotic oxytetracycline is removed by OtrA from the ribosome (Doyle et al., 1991; Mak et al., 2014).

## Multiplicity of Resistance Mechanisms in Producer Organisms

Most producer organisms contain several mechanisms for self-resistance. For example, *S. peucetius* relies on DrrAB to efflux doxorubicin (Li et al., 2014; Brown et al., 2017), DrrC to remove the antibiotic from its target DNA (Prija and Prasad, 2017), and DrrD is possibly used to modify the antibiotic to an inactive form (Karuppasamy et al., 2015). In addition, there is also a serine protease capable of sequestering daunorubicin to prevent its re-entry into the cell following efflux (Dubey et al., 2014). Other examples of producers containing several mechanisms for self-resistance include the following: *Microbispora* ATCC PTA-5024 contains both an efflux pump (MlbJYZ) and a sequestration protein (MlbQ) to protect against NAI-107 (Pozzi et al., 2016); *S. rimosus* has an ABC multi-drug efflux pump (OtrC) (Yu et al., 2012) and an MFS pump (OtrB) for efflux of oxytetracycline (Mak et al., 2014) along with OtrA to protect the ribosome by antibiotic removal (Doyle et al., 1991); *S. fradiae* contains several gene products (TlrA, TlrB, and TlrD) that modify the ribosome to prevent tylosin binding and uses TlrC for efflux (Mak et al., 2014); and *S. chattanoogensis* L10 contains several different efflux pumps for resistance against natamycin (Wang et al., 2017).

## Development of Antibiotic Resistance in Clinical Isolates

Discovery of antibiotics and their development for treatment of infectious diseases is the biggest success story in the history of chemotherapy. However, widespread and indiscriminate use of antibiotics in the last 70 years has led to selection of resistant strains to every antibiotic that has been introduced so far. With the very first antimicrobial agents, such as sulfonamides, resistance was observed soon after in the late 1930s (Davies and Davies, 2010). Even before the widespread use of penicillin in clinical practice, penicillinase was discovered in 1940 in *Staphylococcus aureus* and *Streptococcus pneumoniae* providing evidence that the resistance mechanisms against penicillin were already present in the natural environment (Davies and Davies, 2010; Ogawara, 2016b). Similarly, after the introduction of methicillin (a semi-synthetic penicillin) to treat penicillin-resistant *S. aureus* infections, resistance was once again observed in strains now referred to as MRSA (Methicillin-resistant *Staphylococcus aureus*) (Davies and Davies, 2010). These observations suggest that the use of each and every antibiotic sooner or later results in appearance of resistant strains. This is a testament to the extreme malleability and plasticity of bacterial genomes and their vast potential for adaptability. A high rate of spontaneous mutations and widely prevalent DNA exchange mechanisms in bacteria are critical contributors to the emergence of this phenomenon. According to the Centers for Disease Control and Prevention, antibiotic resistance leads to 23,000 deaths annually in the US alone. Recently, the development of MDR and XDR (extremely drug resistant) strains of *Mycobacterium tuberculosis, S. aureus*, and *Acinetobacter baumannii* have become a cause for serious concern, leaving limited options for the treatment of infectious pathogens carrying these resistance mechanisms. These strains are commonly referred to as ‘superbugs,’ which can be normal human commensal flora that have acquired antibiotic resistance and increased virulence, such as MRSA strains of *S. aureus* and vancomycin resistant enterococci (VRE), or intrinsically resistant environmental bacteria that can become opportunistic pathogens, such as *Pseudomonas aeruginosa* and *A. baumannii* (Wright, 2007; Miller et al., 2014).

## Mechanisms of Antibiotic Resistance in Clinical Isolates

### Intrinsic vs. Acquired Resistance

Intrinsic antibiotic mechanisms are normally chromosome-encoded and include non-specific efflux pumps (which likely evolved as a general response to environmental toxins), antibiotic inactivating enzymes, or mechanisms that serve as permeability barriers (Fajardo et al., 2008; Cox and Wright, 2013). These mechanisms are fixed in the core genetic make-up of an organism. A well-studied example of an intrinsic resistance system is the AcrAB/TolC efflux pump in *Escherichia coli*, which has a very broad substrate specificity and can export different classes of antibiotics, dyes, detergents, and disinfectants (Nikaido and Takatsuka, 2009). Vancomycin resistance in *E. coli* and other Gram-negative bacteria provides another example of intrinsic resistance, which results from the permeability barrier imposed by the outer membrane (Arthur and Courvalin, 1993). Although intrinsic mechanisms confer low level antibiotic resistance in the original host, normal commensal flora or environmental bacteria containing intrinsic mechanisms can become opportunistic pathogens in immunocompromised patients (Wright, 2007). The acquired resistance mechanisms, on the other hand, are generally obtained by horizontal gene transfer (HGT, described later) and include plasmid-encoded specific efflux pumps (such as TetK and TetL of *S. aureus*) and enzymes that can modify the antibiotic or the target of the antibiotic (Bismuth et al., 1990; van Hoek et al., 2011). These mechanisms pose a more serious threat to human health because of a change in the context of the resistance determinant from chromosomal to plasmid-mediated, resulting in their enhanced expression and dissemination (Dantas and Sommer, 2012; Martinez, 2018). A well-documented example of such a phenomenon is mobilization of the chromosomal β-lactamase gene *amp*C to a plasmid resulting in its worldwide dissemination (Dantas and Sommer, 2012).

### Distribution and Function of Resistance Determinants in Clinical Pathogens

Interestingly, the biochemical mechanisms of resistance in clinical isolates are very similar to those found in producer organisms. Moreover, the resistance genes belong to the same functional families as seen in the producers (Benveniste and Davies, 1973; Marshall et al., 1998; Forsberg et al., 2012). However, the distribution, expression, and genetic context of resistance determinants in clinical strains are strikingly different. For example, resistance elements found in producer organisms are embedded in the biosynthesis gene clusters, while in clinical strains they are most often located on plasmids and transposons. For human health reasons, a lot more attention has been given to understanding the molecular and biochemical basis of antibiotic resistance in clinical isolates, and a large number of excellent reviews have been written on this topic (Blair et al., 2015; Chang et al., 2015; Munita and Arias, 2016). Therefore, the section below provides only relevant additional information about each resistance mechanism in clinical strains, allowing the reader to compare and contrast our understanding of these determinants in clinical strains vs. the producer organisms while providing a more complete picture of the field of antibiotic resistance. Where available, examples of antibiotic resistance genes/mechanisms in non-producing environmental bacteria are also provided, and their possible relationships with determinants in clinical strains are discussed (Table 2).

**Table 2:** Antibiotic resistance mechanisms in clinical isolates.

Mechanism of resistance	Intrinsic resistance	Gene location	Reference	Acquired resistance	Gene location	Reference
Antibiotic modification/degradation	AME AAC(2′)-Ia	Chromosome *P. stuartii* (*aac(2*′*)-Ia*)	Macinga and Rather, 1999	AME AAC(6′)-Ib’	MGE *P. aeruginosa* (*aac(6*′*)-Ib*′ integron)	Ramirez and Tolmasky, 2010
	β-lactamase AmpC	Chromosome *E. coli* (*bla*_AmpC_)	Jacoby, 2009	β-lactamase TEM-3	MGE *K. pneumoniae* (*bla*_TEM-3_ plasmid)	Paterson and Bonomo, 2005
Antibiotic efflux	RND AcrAB/TolC (MDR)	Chromosome *E. coli* (*acr*AB/*tol*C)	Thanassi et al., 1997	SMR QacC (MDR)	MGE *S. aureus (qac*C plasmid)	Schindler and Kaatz, 2016
	MFS NorA (MDR)	Chromosome *S. aureus* (*nor*A)	Schindler and Kaatz, 2016	MFS TetK, TetL (tetracycline)	MGE *S. aureus* (*tetK*, *tetL* plasmid)	Bismuth et al., 1990; van Hoek et al., 2011
Antibiotic sequestration with special proteins	Sequestration Lipocalin (polymyxin B, rifampicin, norfloxacin, ceftazidime)	Chromosome *B.cenocepacia (bcn*A)	Sabnis et al., 2018	Sequestration BLMS, BMLT (bleomycin)	MGE *S. aureus (ble* on plasmid) *E. coli* (*ble* on *Tn*5)	Sugiyama et al., 1995; Kumagai et al., 1999
Antibiotic target modification	Low affinity PBP PBP1	Chromosome*M. leprae* (*pon*1)	Basu et al., 1996	Low affinity PBP PBP2a	MGE *S. aureus* (*mec*A in SCC*mec*)	Fishovitz et al., 2014
	Peptidoglycan remodeling (GPAs) VanC, VanXY_C_, VanT_C_, VanR_C,_ VanS_C_	Chromosome *E. gallinarum* (*van*C cluster)	Binda et al., 2014; Miller et al., 2014	Peptidoglycan remodeling (GPAs) VanRS, vanHAXYZ	MGE *E. faecalis* (*van*A cluster *Tn*1546 on plasmid)	Binda et al., 2014; Miller et al., 2014
	23S rRNA Methylation (MLS) ErmMT	Chromosome*M. tuberculosis* (*erm*MT)	Buriankova et al., 2004	23S rRNA methylation (MLS) ErmC	MGE *S. aureus* (*erm*C plasmids)	Roberts, 2008
	16S rRNA Methylation (AGs) EfmM	Chromosome *E. faecium (efm*M)	Galimand et al., 2011	16S rRNA methylation (AGs) ArmA	MGE *K. pneumoniae* (*arm*A on plasmid)	Doi et al., 2016
Antibiotic target bypass	Overproduction DHFR (TMP)	Chromosome *E. coli* (mutation in promoter of *dhfr*)	Huovinen, 2001; Munita and Arias, 2016	Low affinity DHPS (sulfonamide)	Chromosome *N. meningitidis* (*dhps)* by transformation	Radstrom et al., 1992
Antibiotic target protection	Antibiotic removal LsaA (lincosamide and streptogramin A)	Chromosome*E. faecalis* (*lsa*)	Murina et al., 2018	Antibiotic removal TetO (tetracycline)	MGE *C. jejuni* (*tet*O) plasmid, transposon	Munita and Arias, 2016


*AME, aminoglycoside modifying enzyme; AAC, N-acetyltransferase; MGE, mobile genetic element; RND, Resistance-Nodulation-Division; SMR, Small Multidrug Resistance; MFS, Major Facilitator Superfamily; MDR, Multidrug resistance; PBP, penicillin-binding protein; GPAs, glycopeptide antibiotics; MLS, macrolides, lincosamides, and streptogramins; AGs, aminoglycosides; DHFR, dihydrofolate reductases; DHPS, dihydropteroic acid synthase; TMP, trimethoprim.*

#### Antibiotic Modification

As seen in producers, antibiotic modification is commonly used as a resistance mechanism for aminoglycosides in pathogenic strains. Multiple types of AMEs (∼100), including a fusion enzyme containing both AAC and APH activities, have been identified in both Gram-positive and Gram-negative bacteria (Schwarz et al., 2004; Ramirez and Tolmasky, 2010), and a detailed nomenclature has been developed (Ramirez and Tolmasky, 2010; Becker and Cooper, 2013). While these genes are commonly located on the mobile genetic elements (MGEs) in clinical bacteria, chromosomal determinants for AMEs have also been found in a large number of environmental bacteria, including *Providencia* and *Acinetobacter* species (Macinga and Rather, 1999; Yoon et al., 2014), which are considered to be the source of acquired determinants found on MGEs in pathogenic strains. Of the known AMEs, AACs are the most prevalent in clinical strains, and the AAC (6′) enzymes, which acetylate at the 6′ position of the aminoglycoside scaffold, have been studied in detail. In spite of the presence of a conserved fold, these enzymes exhibit significant sequence, structural, and functional diversity, again implying convergent evolution of these enzymes from distinct housekeeping cellular proteins (Stogios et al., 2017). Indeed, in the environmental bacteria *Providencia stuartii*, physiological function of the chromosomally encoded AAC(2′)-Ia enzyme is thought to be acetylation and recycling of peptidoglycan although it can also acetylate aminoglycosides (Macinga and Rather, 1999). Therefore, aminoglycosides may be ‘accidental’ substrates for these enzymes because of their similarity to cellular substrates containing amino sugars (Macinga and Rather, 1999). These studies further illustrate the plasticity of antibiotic modification enzymes (Fong et al., 2011; Stogios et al., 2017), as discussed previously for the producers. In addition to AMEs, multiple CAT enzymes have been identified in both Gram-positive and Gram-negative bacteria, which have been extensively reviewed (Schwarz et al., 2004).

A third type of modification/degradation enzyme used by clinical bacterial strains is β-lactamase. While the role of β-lactamases in producer bacteria is still debatable, they are known to play a critical role in β-lactam resistance in Gram-negative clinical bacteria. Gram-positive bacteria instead prefer PBP-based resistance mechanisms, likely due to differences in the architecture of the cell wall/envelope between the two types of bacteria. More than 1000 β-lactamases have been identified from clinical isolates, and this number continues to grow because of the ever-new mutations in the active site allowing it to adapt to newer β-lactams. An example is the evolution of TEM-3, which can degrade 3rd generation cephalosporins, placing it into the category of ESBLs (Extended Spectrum β-lactamases) (Paterson and Bonomo, 2005), suggesting rapid evolution of β-lactamase genes in clinical strains. Most β-lactamase genes are carried on MGEs facilitating their rapid spread through populations; however, some β-lactamase genes are also found in chromosomes of members of the *Enterobacteriaceae* family where they are poorly expressed and function as silent genes. Once again, it is speculated that, as in the case of AMEs, β-lactamases may also perform dual functions, including housekeeping and antibiotic resistance (Martinez, 2018). An interesting set of studies indeed suggest that the biological function of β-lactamases may be peptidoglycan recycling (Wiedemann et al., 1998; Macinga and Rather, 1999), although their mobilization to a plasmid results in high expression and high levels of antibiotic resistance (Jacoby, 2009; Dantas and Sommer, 2012).

#### Antibiotic Efflux

The second major mechanism of antibiotic resistance in clinical strains involves decreased permeability and/or efflux of the antibiotic. Decreased permeability is important for Gram-negative bacteria because of the presence of the outer membrane, which forms a permeability barrier and offers an intrinsic mechanism for protection against hydrophilic antibiotics and other antimicrobial agents, such as vancomycin (Nikaido, 2003). Mutations in the porin genes and/or changes in their expression have been shown to further impact the susceptibility of Gram-negative bacteria to hydrophilic antibiotics (Li et al., 2012). In addition, many types of active efflux pumps have been described in Gram-positive and Gram-negative bacteria, which generally belong to one of the five families: ABC, MFS, RND (Resistance-Nodulation-Division), MATE (Multidrug and Toxin Extrusion), and SMR (Small Multidrug Resistance) (Sun et al., 2014; Schindler and Kaatz, 2016). Of these, only ABC proteins use ATP as a source of energy, while the other four families couple transport of substrates to ion gradients. Normally transport proteins carry out import or export of only one specific substrate (for example, Tet proteins belonging to the MFS family). However, examples of multidrug/polyspecific exporters have been found in each of these five families (Poole, 2005; Schindler and Kaatz, 2016), suggesting that polyspecificity is widely distributed and must be an ancient phenomenon.

Genes encoding antibiotic efflux pumps can be either intrinsic or acquired. Examples of intrinsic genes include *acr*AB/*tol*C in *E. coli*, *nor*A in *S. aureus*, and *lmr*A in *Lactococcus lactis*. Of these, the best understood system is the tripartite RND pump AcrAB/TolC. Although this system carries out efflux of a very broad spectrum of compounds, its biological function is believed to be export of bile salts in *Enterobacteriaceae* (Thanassi et al., 1997; Martinez, 2018). The RND pumps are unique in that they bridge the inner and outer membranes through a fusion protein (AcrA in this case) and bring about export of antibiotics from the inside to the outside in a single step. The acquired antibiotic efflux determinants, often found on MGEs in clinical isolates, are exemplified by many different types of *tet* genes (at least 22 have been identified) located on plasmids in both Gram-negative and Gram-positive bacteria (Roberts, 2005). Interestingly, RND pumps can act synergistically with the simple Tet pump proteins (MFS family), resulting in a significant increase in the minimum inhibitory concentration for tetracycline (Lee et al., 2000). This likely occurs when tetracycline exported to the periplasm by a Tet protein can be captured by the RND pump and exported to the outside (Nikaido and Takatsuka, 2009), illustrating how acquired resistance mechanisms can be augmented by the intrinsic mechanisms potentially resulting in major implications in the clinic.

#### Target Modification/Bypass/Protection

A large number of target replacement and protection mechanisms are also found in clinical isolates. The classical example of target modification is seen in MRSA strains where resistance to β-lactams is conferred by an exogenous PBP, known as PBP2a, whose transpeptidase domain is insensitive to the action of several different β-lactams. Acquisition of PBP2a facilitates bypass of the original sensitive target, however, since it does not contain the transglycosylase activity it functions together with the transglycosylase domain of the native PBP2 to perform cross-linking reaction in the presence of β-lactams. PBP2a is coded by the *mec*A gene, which is located on a large MGE called SCC*mec* (Staphylococcal chromosomal cassette) in *S. aureus*. Many different types of SCC*mec* cassettes have been described, which contain varying numbers of accompanying resistance elements (Fishovitz et al., 2014; Liu et al., 2016). Another example of target modification is vancomycin resistance, which results from acquisition of the *van* gene cluster and is commonly a problem in enterococci (Miller et al., 2014). Of the many known types of *van* clusters, *van*A and *van*B, in particular, are a problem in clinical strains as they occur on MGEs. The similarities in the sequence and arrangement of *van* genes in producer and clinical strains suggest that they are evolutionarily linked.

Other target modification examples in clinical strains include point mutations or enzymatic alteration of the target (Munita and Arias, 2016). For examples of point mutations in the target, see (Hooper, 2002; Floss and Yu, 2005). Enzymatic alteration of the target is best understood in the case of macrolide resistance conferred by a large group of erythromycin ribosomal methylation (*erm*) genes. These enzymes methylate a specific adenine in the 23S rRNA (Weisblum, 1995). The *erm* genes in clinical strains are present on mobile genetic elements and are widespread among both Gram-positive and Gram-negative bacteria (Roberts, 2008). Significant similarities between the methylation enzymes found in the clinical isolates and the producers have been observed, suggesting a common ancestral origin (Uchiyama and Weisblum, 1985; Doi et al., 2016). Finally, known examples of target protection in clinical strains include the Tet(M) and Tet(O) proteins commonly encoded by genes located on MGEs in *S. aureus*. Interestingly, these proteins are homologous to the elongation factors EF-G and EF-Tu, and their binding to the ribosome facilitates removal of tetracycline in a GTP-ase activity-dependent manner (Burdett, 1996; Trieber et al., 1998).

Based on the discussion above, it is evident that our understanding of the distribution and function of resistance determinants in clinical isolates is much more advanced as compared to the producer organisms. It may also be concluded that many (or most) of the antibiotic resistance mechanisms in producers, and possibly all organisms, appear to have been repurposed from housekeeping/cellular functions or the intrinsic resistance mechanisms. Indeed, it is the incorporation of such determinants into MGEs in pathogens that poses a serious threat to human health.

## Origin of Antibiotic Resistance in Clinical Isolates

Where do antibiotic resistance genes in the clinic come from? This question continues to puzzle scientists and clinicians. The idea that resistance genes in pathogens may be acquired from antibiotic producer organisms by horizontal transfer was originally proposed in the 1970s (Benveniste and Davies, 1973). It was based on the observation that the aminoglycoside-modifying enzymes found in actinomycetes exhibit biochemical activities similar to the enzymes found in pathogenic strains. Another striking example of a strong connection between antibiotic resistance genes in clinical isolates and those found in antibiotic producing bacteria is provided by the *van*HAX genes, which show considerable protein sequence similarity as well as a conserved arrangement and organization of genes within the cluster (Barna and Williams, 1984; Marshall et al., 1998).

**FIGURE 2:**
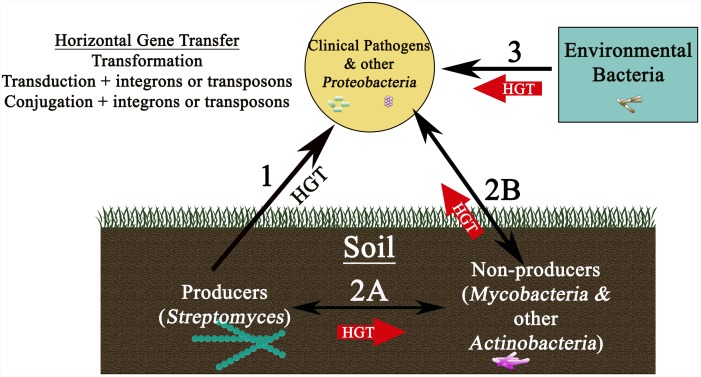
Schematic showing reservoirs of antibiotic resistance genes found in nature and various pathways for their movement to the clinic. Transfer of resistance genes to clinical isolates could occur by a variety of routes (shown by arrows), each using horizontal gene transfer mechanisms potentially involving plasmids, integrons, or transposons. While direct transfer of resistance determinants from producers in the soil to clinical strains is possible (Route 1), a more likely route may first involve movement from the producer soil bacteria to non-producer soil bacteria (for example *Mycobacterium* species) (Pang et al., 1994) (Route 2A), followed by transfer to clinical pathogens through several carriers (Route 2B). Another, possibly more important route, could involve direct transfer from environmental bacteria (found in bodies of water, aquaculture, livestock animals, wildlife, and plants) to clinical isolates (Route 3). Routes 2 and 3 are shown as thick red arrows, implying greater probability of these pathways for dissemination of resistance genes to clinical strains.

Despite strong indications that transfer from producer organisms to the pathogenic strains might occur (Figure 2, Route 1) a direct link between producers and pathogens has, however, been hard to establish, and very rarely have the resistance genes of pathogens been tracked back to the producers. This is primarily due to the fact that resistance genes in producers show high sequence divergence and a very different G+C content as compared to determinants in pathogens even when they use similar mechanisms (Forsman et al., 1990; Marshall et al., 1998). Altogether, these observations suggest an evolutionary link between determinants of producers and pathogens but not necessarily a direct recent gene transfer from the producers (Forsman et al., 1990; Marshall et al., 1998; Aminov and Mackie, 2007). Nevertheless, transfer from producers could have occurred a long time ago through a series of closely related carriers; for example, first transfer to closely related non-producing actinomycetes in the soil (Figure 2, Route 2A) and then finally to proteobacteria and distant pathogenic strains (Marshall et al., 1998) (Figure 2, Route 2B). The longer time horizon in this case could explain a very different G+C content in the two groups of organisms.

An alternative school of thought and a growing body of recent literature, however, now seem to suggest that resistance genes found in non-producer environmental bacteria may have played a more important role in shaping the evolution of antibiotic resistance in pathogens (Figure 2, Route 3) (Aminov and Mackie, 2007). Indeed, resistance genes are much more widespread in environmental non-pathogenic microbial populations than was originally believed (D’Costa et al., 2006; Nesme et al., 2014; Surette and Wright, 2017). In an interesting study, which tested 500 *Streptomyces* strains enriched and isolated from soil against 21 antibiotics (including natural, semisynthetic, synthetic as well as recently introduced antibiotics), surprisingly all strains were multidrug resistant to 7 or 8 of the 21 tested antibiotics (D’Costa et al., 2006), suggesting widespread resistance mechanisms among modern organisms. The genome sequence analyses carried out in recent years have also shown that not only are the intrinsic resistance mechanisms widely prevalent in all microbes (Fajardo et al., 2008; Cox and Wright, 2013), but that homologs of the resistance determinants of clinical isolates are commonly present in non-pathogenic Gram-positive and Gram-negative bacteria (Seoane and Garcia Lobo, 2000; Mukhtar et al., 2001; Sugantino and Roderick, 2002). Finally, there is also strong evidence showing that the antibiotic resistance gene sequences are ancient and predate the use of antibiotics (D’Costa et al., 2011; Bhullar et al., 2012; Warinner et al., 2014; Perron et al., 2015; Kashuba et al., 2017). Analysis of microbial DNA isolated from the dental plaque of ancient human remains showed the existence of gene sequences homologous to those conferring resistance to β-lactams, aminoglycosides, macrolides, tetracycline, and bacitracin in clinical strains (Warinner et al., 2014; Olaitan and Rolain, 2016). In another study, metagenomic analysis of ancient DNA derived from 30,000-year-old permafrost showed the presence of homologs of *tet*M, *van*X, and *bla* genes (D’Costa et al., 2011). Interestingly, the *van*HAX cluster in permafrost DNA exhibited the same invariant organization as seen in modern vancomycin resistant isolates, confirming that these genes predate the use of antibiotics. Other similar studies showing prevalence of resistance determinants in ancient samples, or isolated caves, are also available (Bhullar et al., 2012; Perron et al., 2015; Kashuba et al., 2017). Together these findings suggest that there is a continuum of resistance genes present in the environmental, producer, and pathogenic organisms, leading to the concept of ‘resistome’ which is described as the collection of antibiotic resistance genes found in all microorganisms (Wright, 2007). Therefore, it is proposed that to get a full understanding of the origin of resistance, one must consider the pan-microbial genome consisting of antibiotic producers, pathogens, cryptic genes, and precursor genes (Wright, 2007; Nesme and Simonet, 2015).

Overall, it is safe to conclude that both producer and non-producing environmental organisms represent rich pools of resistance genes which could potentially be mobilized to the clinically relevant strains, leading to the question ‘is the evidence for transfer of resistance determinants using any of the routes proposed in Figure 2 actually available’? Albeit limited in number, a few reports of direct genetic exchange from producer to non-producer organisms and from environmental organisms to clinical pathogens are indeed available. In one report, *otr*A and *otr*B gene sequences, found in the oxytetracycline biosynthesis cluster in *Streptomyces*, were identified in mycobacteria variants (Pang et al., 1994). Mycobacterium is closely related to *Streptomyces*, and both are commonly found in the soil, therefore the transfer of *otr*A and *otr*B to mycobacteria suggests their role as potential carrier organisms in the soil. Interestingly, the same study also provided evidence for the presence of *S. aureus* tetracycline resistance genes Tet(K) and Tet(L) in *Streptomyces* and mycobacteria variants. The sequences isolated from these variants were almost identical to the *S. aureus* genes and had a G+C content of only 35% as compared to the 70% G+C content normally seen in *Streptomyces* and mycobacteria, which is a strong indication that these resistance elements originated from low G+C Gram-positive bacteria (Pang et al., 1994). This study therefore shows that resistance genes can move back and forth between producer and non-producer organisms providing support for Route 2A (Figure 2). In another study, bioinformatics analysis was used to obtain evidence for recent inter-phylum transfer of chloramphenicol and lincomycin efflux genes *cmx* and *lmrA* from *Actinobacteria* to *Proteobacteria* (Jiang et al., 2017), possibly also occurring through Route 2B, which may be followed by transfer of these genes to clinical isolates (Figure 2). The proposed mechanism for such inter-phylum exchange is discussed in (Jiang et al., 2017) and briefly described in Section “Role of HGT in Transfer of Antibiotic Resistance Genes” in this article.

The most compelling evidence of recent transfers from non-pathogenic environmental bacteria to clinical strains (Figure 2, Route 3) comes from three independent reports (Dantas and Sommer, 2012; Forsberg et al., 2012). First report showed that the CTX-M ESBL gene found on plasmids in pathogenic bacteria worldwide is almost identical to CTX-M gene found in the genome of non-pathogenic environmental *Kluyvera* species (Humeniuk et al., 2002; Canton and Coque, 2006), suggesting recent transfer of the gene to clinical strains. The second report shows that the quinolone resistance determinant *qnr* located on a conjugative plasmid in *Klebsiella*, originated from the genome of non-pathogenic environmental *Vibrio* and *Shewanella* species (Poirel et al., 2005). And yet another example provides evidence for transfer of the *aph*6 gene, which codes for Aph (3′)-VI amikacin modification enzyme, from the chromosome of the environmental *Acinetobacter guillouiae* to a plasmid in *A. baumannii* and then to members of *Enterobacteriaceae* family and to *Pseudomonas* species (Yoon et al., 2014). These examples provide definitive evidence of genetic transfer from environmental organisms and also illuminate how an intrinsic resistance gene located in the genome of a non-pathogenic organism can result in a pandemic when mobilized to a conjugative plasmid or a phage and transferred to a clinically relevant strain. Overall, these examples suggest that both producer and non-producer environmental bacteria play a role in dissemination of resistance genes although recent direct transfers to clinical strains seem to have mainly occurred from non-producer environmental bacteria.

## Role of HGT in Transfer of Antibiotic Resistance Genes

Transfer of antibiotic resistance determinants between bacterial populations occurs by genetic exchange mechanisms involving transformation with free DNA, transduction by bacteriophages, or conjugation involving plasmids (Wright, 2007; Hu et al., 2017), collectively referred to as the HGT mechanisms. All three HGT mechanisms are widely used in nature, although certain species of bacteria tend to employ one mechanism more heavily over the others (Barlow, 2009). For example, streptococci can become naturally competent and thus participate effectively in transformation, whereas enterobacteria commonly use conjugative plasmids for exchange of genetic information. Transformation is best characterized in Gram-positive *Streptococcus pneumoniae* and *Bacillus subtilis* although many Gram-negative bacteria also become competent (Johnston et al., 2014). The factors that control competence generally include the nutritional status of the bacterium (Claverys et al., 2006) and environmental stressors, such as antibiotics or DNA damaging agents (Prudhomme et al., 2006). Although the physiological role of transformation is still debated, its main purpose is believed to be DNA repair or genetic diversification to enhance adaptability (Johnston et al., 2014). Indeed, transformation seems to have played an important role in evolution of antibiotic resistance strains of *Streptococcus* and *Neisseria*. For example, it is thought that the persistence of penicillin resistance in *S. pneumoniae* may be related to the high frequency of natural transformation in this organism (Hoffman-Roberts et al., 2005). Transformation of *Neisseria gonorrhoeae* with DNA from resistant commensal *Neisseria flavescens* is believed to have resulted in generation of a mosaic *pen*A variant that confers resistance to β-lactams in clinical isolates (Spratt, 1988; Spratt et al., 1992). Mosaic variants of antibiotic resistance genes have also been reported in several *Streptococcus* species, implying the role of transformation in incorporating sections of foreign DNA (von Wintersdorff et al., 2016).

Transduction is believed to play a major role in evolution of resistance in *S. aureus*, although it has been shown to occur in many bacteria at a low frequency ranging between 10^-6^ and 10^-9^ transductants/plaque-forming-unit (Ubukata et al., 1975; Mazaheri Nezhad Fard et al., 2011; Varga et al., 2012). In *S. aureus*, which exhibits high strain variability and carries a large accessory genome consisting of phages, plasmids, transposons, genomic islands, and SCC_mec_ (most of which carry resistance genes), it is generally accepted that HGT in general, and transduction in particular, play a major role in antibiotic resistance gene transfer (Haaber et al., 2017). Indeed, moderate rates of transfer (about 10^-5^ or 10^-6^) of genes for penicillinase, metallo β-lactamase, and tetracycline resistance by transducing phages have been reported in *S. aureus* (Varga et al., 2012; Lee and Park, 2016; Varga et al., 2016). However, transduction of even the small SCC_mecs_ (20–25 kb in size) from MRSA strains of *S. aureus* to methicillin-sensitive strains was shown to occur at low frequencies (10^-9^ to 10^-10^) (Scharn et al., 2013). Another study, which used qPCR to quantify *S. aureus* genes in viral particles, showed the presence of parts of the SCC_mec_ element (specifically *mec*A and *ccr*A1) in phage particles at relatively high frequency of about 10^-4^ (Maslanova et al., 2013). Quantitative studies, however, do not take into consideration the transmission capability of the particles, therefore they likely reflect an overestimation of the transduction frequency (Torres-Barcelo, 2018). Interestingly, other resistance and virulence genes of *S. aureus* associated with special MGEs referred to as PICIs (phage-induced chromosomal islands), which include SaPIs (*S. aureus* pathogenicity islands), are known to be transduced by bacteriophages at remarkably high frequencies approaching 10^-1^ (Chen and Novick, 2009; Penadés and Christie, 2015). These islands include many antibiotic resistance genes, suggesting that transduction may contribute significantly to variability and evolution of resistance in *S. aureus* (Novick et al., 2010). Interspecies and intergeneric transfer of SaPI elements has also been shown to occur between *S. aureus, S. epidermidis*, and even *Listeria monocytogenes*, showing a broader host range of staphylococcal phages (Maiques et al., 2007).

In general, however, because of the difficulty in detecting recombination events outside of the laboratory, the contribution of either transformation or transduction in transferring resistance genes in the clinic or the environment remains unclear. Nevertheless, certain environments considered to be hot-spots for genetic exchange, such as sewage and wastewater treatment plants, hospital effluents, aquaculture, agricultural and slaughterhouse waste, are prime locations for exchange events because of the high density of bacteria, phages, and plasmids in these settings (Kenzaka et al., 2010; von Wintersdorff et al., 2016). In one study, qPCR analysis showed that *bla*_TEM_, *bla*_CTX-M_, and *mec*A were indeed present in phage particles isolated from sewage samples (Colomer-Lluch et al., 2014). Other reports showing the prevalence of phage carrying *bla*_TEM_ and *bla*_CTX-M_ genes in soil, water, and sewage are also available (Balcazar, 2014; Larranaga et al., 2018; Mohan Raj et al., 2018). When combined with high selection pressure in these environments, resulting from the presence of sub-inhibitory concentrations of antibiotics, metals, and toxic materials, which can lead to induction of competence (Prudhomme et al., 2006) as well as induction of prophages (Motlagh et al., 2015), it further enhances the possibility of HGT by these two mechanisms. Overall, these reports suggest that the original transfer of CTX-M from *Kluyvera* to the clinic pathogens, referred to in Section “Origin of Antibiotic Resistance in Clinical Isolates,” might have been mediated by bacteriophages. Other settings suitable for genetic exchange via transduction also include the colonized human or animal host (McCarthy et al., 2014; Stanczak-Mrozek et al., 2015), gut microbiome (Modi et al., 2013), and biofilms (Resch et al., 2005). A recent report describing the phenomenon of auto-transduction in *S. aureus* provides further strong support for the important role of phages in delivering antibiotic resistance genes to the host bacteria (Haaber et al., 2016). Using *in vitro* and *in vivo* virulence model, this study by Haaber et al. (2016) demonstrates how phages released from a subpopulation of lysogenic cells can lyse other phage-sensitive cells in the same environment, recruit beneficial genes from the killed competitors, and reintroduce these genes into the remaining lysogenic host cells, resulting in genetic diversity.

Plasmid-mediated conjugation as a gene transfer mechanism is, however, still considered to be far more prevalent in disseminating resistance genes in nature than either transformation or transduction. Plasmids are capable of autonomous replication, and they carry genes for resistance against all major classes of antibiotics. In fact, plasmids can carry a collection of resistance genes as part of transposons, thus simultaneously conferring resistance to several classes of antibiotics and metal ions (Nikaido, 2009). Moreover, they can transfer genes over long genetic distances to different species, genera, and even kingdoms depending on the host range of the plasmid. Using mathematical modeling analysis, one study recently showed that conjugation may be 1000-fold more common than transduction as a resistance gene transfer mechanism (Volkova et al., 2014). Since gene transfer by conjugation can be easily tracked by DNA sequencing and PCR-based approaches, there is sufficient evidence for its contribution to worldwide dissemination of antibiotic resistance determinants both in community and hospital environments (Carattoli, 2013). Some of the most successful known plasmids are the ones that have resulted in the spread of carbapenemase, *bla*_CTX-M_ ESBL, and quinolone resistance genes among Gram-negative bacteria over very large geographical distances (Carattoli, 2013). In Gram-positive bacteria, other DNA elements, known as conjugative transposons or integrative conjugative elements (ICEs), can also mediate conjugation. These elements integrate into the chromosome but contain the ability to excise and transfer themselves by conjugation. ICEs often carry resistance genes, for example Tn*916* family members that encode tetracycline resistance (Roberts and Mullany, 2011). The known conditions for resistance gene transfer by conjugation include high density settings, such as the human or animal gut, biofilms, hospitals, and co-infection conditions (Weigel et al., 2003; Savage et al., 2013; Huddleston, 2014; Andersson and Hughes, 2017). Although some resistance determinants have been plasmid-associated for a long time (Barlow and Hall, 2002), others are mobilized to plasmids from chromosomes, and the rate at which these genes are being mobilized has increased since the widespread use of antibiotics about 70 years ago (Barlow et al., 2008). Another worrisome emerging trend is the clustering of antibiotic resistance genes on plasmids, perhaps as a response to selective pressures in the environment. A well-characterized mechanism of clustering is provided by the *S. aureus* conjugative plasmid pSK41 that contains an insertion sequence IS*257*, which promotes capture of small resistance plasmids (Haaber et al., 2017).

All three HGT mechanisms are subject to limitations imposed by the host range of the incoming plasmid or the phage, the restriction modification systems of the host, ability to form cell-to-cell contacts, fitness cost of acquiring a new gene, as well as the ability of the incoming DNA to recombine with the host DNA (Thomas and Nielsen, 2005; Domingues et al., 2012). Further, the ability of a mobile genetic element to establish in a population also depends on whether it can replicate autonomously and therefore get vertically transmitted. The most successful conjugative plasmids, such as the incompatibility group IncP, have a broad host range (Davies and Davies, 2010), which facilitates their transfer to and maintenance in distantly related phyla (Klumper et al., 2015). The ability of MGEs or DNA to persist in the environment also determines success of HGT. For example, while cell-to-cell contact is essential for conjugation, it provides better protection to DNA. On the other hand, naked DNA is vulnerable to being degraded quickly, which reduces the time period during which it remains intact to successfully encounter a competent cell. DNA packed in a phage particle is more protected than naked DNA, although the narrow host range of a phage may determine if it will be in the gene pool long enough to infect a suitable host (von Wintersdorff et al., 2016).

In spite of the limitations, bacterial genome sequencing efforts have made it abundantly clear that the HGT mechanisms have had a major impact on evolution of bacterial populations (Nakamura et al., 2004; Andam et al., 2011; McDonald and Currie, 2017). Our knowledge of the actual steps and carriers involved in moving resistance genes from environmental and producer organisms to the clinic, or from the chromosome to the MGEs, is, however, still rather limited. In each of the examples described in Section “Origin of Antibiotic Resistance in Clinical Isolates,” exchange was facilitated by conjugative plasmids (Humeniuk et al., 2002; Poirel et al., 2005; Yoon et al., 2014) or by the presence of resistance genes on transposons (Brisson-Noel et al., 1988). It is not clear, however, why and how resistance genes are captured or transferred from chromosome to the plasmids. In addition to the role of insertion sequences and transposons, mobilization of resistance genes may also be greatly aided by the presence of integrons. While they are not self-mobile, they can be mobilized to plasmids or phages by transposons, thus gaining the ability to move between cells by HGT. Integrons typically contain three genetic elements, which include a gene for site-specific recombination (*Int*I), a site-specific recombination site (*att*I), and a promoter upstream of the *att*I site used for expression of the recruited gene cassette (often containing resistance determinants) (Domingues et al., 2012). Thus they are able to exchange and/or recruit gene cassettes by site-specific recombination between the *att*C site on the cassette and the *att*I site on the integron, or they can excise gene cassettes by site-specific recombination, therefore conferring the ability on the host to rearrange resistance and virulence determinants (Gillings, 2014). Class 1 integrons found on MGEs, in particular, are widely distributed in clinical settings and are often associated with carrying and spreading antibiotic resistance genes (Naas et al., 2001; Li et al., 2017). A rather large pool of circular gene cassettes containing the *att*C site and the promoter-less resistance determinants for almost all classes of antibiotics used clinically are also known to exist in bacteria (Partridge et al., 2009). These genes become functional after the cassettes are incorporated and expressed from the promoter sequence in the integron.

Recently, a novel ‘carry-back’ mechanism for inter-phylum exchange of genes was also proposed (Jiang et al., 2017). In this mechanism, conjugation mediated by a broad-host range conjugative plasmid (Klumper et al., 2015) may transfer a carrier sequence of DNA (a fragment from a widely spread class 1 integron In4) from *Proteobacteria* to *Actinobacteria*, followed by recombination, resulting in actinobacterial DNA flanked by proteobacterial DNA. Dead actinobacteria cells would release the actinobacterial DNA flanked by proteobacterial DNA into the environment, and proteobacteria can take up this DNA by transformation and incorporate into their genome using homologous recombination. Using such a mechanism, *cmx* and *lmr*A genes are believed to have been recently transferred from *Actinobacteria* to *Proteobacteria* with the help of the broad-host range conjugative plasmids and integrons (Jiang et al., 2017). Once these genes are transferred to proteobacteria, it is easy to envision their transfer to pathogenic bacteria which also mostly belong to the phylum *Proteobacteria*. Indeed the *Proteobacterial* Cmx protein identified in clinical isolates was found to be 52% identical to the self-resistance protein from producer *S. venezuelae*, and the *cmx* gene was found to be 99% identical to genes from many non-*Streptomyces* actinobacteria, including *Corynebacterium* species, suggesting recent inter-phylum transfer from *Actinobacteria* to *Proteobacteria* following Route 2B.

## Enrichment of Antibiotic Resistance Genes

By now it is well-recognized that the environment itself plays an important role in the acquisition of antibiotic resistance by pathogenic organisms. This process is envisioned to go through four stages: emergence of novel resistance genes, mobilization, transfer to pathogens, and dissemination. While emergence and mobilization events likely occur all the time, environmental factors, such as selective pressure, fitness cost, and dispersal, determine whether these events actually result in establishing novel genes in populations (Bengtsson-Palme et al., 2018). Of these, selection is perhaps the single most important factor which plays a critical role in maintenance of resistance genes/MGEs at each stage of the acquisition process described above. What creates selective pressure strong enough to promote persistence and longevity of resistance genes? Antibiotic producers present one such scenario where resistance genes can be selected naturally in a competitive environment, thus preserving the pool of resistance genes in that niche (Laskaris et al., 2010). The most important source of selective pressure, however, is the widespread and indiscriminate usage of antibiotics by humans, which results in dominance of resistant and multiply resistant strains of bacteria not only among human pathogens but also in environments where human activities (such as antibiotic manufacturing facilities) result in pollution with antibiotics (Larsson, 2014). Other settings, considered to be hot-spots (described in section “Role of HGT in Transfer of Antibiotic Resistance Genes”), where human-associated and environmental bacteria co-exist, also provide significant opportunities for exchange of resistance genes as well as selection for resistance (Bengtsson-Palme et al., 2018). Such environments are ideal not only for transfer of resistance genes to pathogens, but they can also result in transfer of resistance from pathogens to environmental bacteria or opportunistic pathogens, resulting in persistence and possible reemergence of resistance genes in the future (Ashbolt et al., 2013; Martínez et al., 2014; Bengtsson-Palme et al., 2018). Recent studies have shown that antibiotic concentrations significantly below the minimum inhibitory concentration for sensitive bacteria can be selective (Gullberg et al., 2011, 2014). Moreover, other contaminants, such as heavy metals, can also co-select for antibiotic resistance (Pal et al., 2015; Andersson and Hughes, 2017).

There is indeed evidence that selective pressure caused by human activities in the last 70 years has resulted in a significant enrichment of resistance genes in bacterial populations. One study compared pre-antibiotic era microbes with modern environmental bacteria in archived soils collected from 1940 to 2008 in the Netherlands and showed that genes conferring resistance to tetracycline, erythromycin, and β-lactams increased in abundance over time (Knapp et al., 2010). Interestingly, an increased rate of mobilization of β-lactamase genes from the chromosome to the plasmids was also reported (Barlow et al., 2008). A novel hypothesis advanced recently suggests that the use of antibiotics may provide a strong selection for ‘capture’ of antibiotic resistance genes by mobile genetic elements (including plasmids, transposons, and integrons) and acting as a strong force in shaping evolution of microorganisms (Gillings, 2014; Surette and Wright, 2017). Other reports also suggest that antibiotic selection promotes competence in *S. pneumoniae* (Prudhomme et al., 2006), induction of prophages in *S. aureus* (Goerke et al., 2006), and enrichment of antibiotic resistance genes in phages present in the gut microbiome (Modi et al., 2013), all processes that could increase the rate of HGT. Interestingly, a more recent study showed that the ratio of transducing particles to virulent phages varies upon induction by sub-inhibitory concentrations of different antibiotics, suggesting that antibiotics affect packaging of genes into phage particles (Stanczak-Mrozek et al., 2017). Antibiotic exposure has also been shown to result in increased rates of mutations and recombination as well as an increase in integrase activity (Maiques et al., 2006; Lopez et al., 2007; Blazquez et al., 2012), thus compounding the multiple effects that excessive usage of antibiotics can have on emergence and enrichment of antibiotic resistance in bacterial populations. In conclusion, mitigation strategies focused on limiting selective pressure, for example by reducing unnecessary usage of antibiotics and avoiding settings which select for and promote persistence, are needed to prevent further recruitment of novel resistance genes into pathogens.

## Conclusion, Research Gaps, and Future Directions

Antibiotic producing bacteria of the genus *Streptomyces* as well as non-pathogenic environmental bacteria are important reservoirs of antibiotic resistance determinants. These determinants may be transferred to clinical strains by a variety of HGT mechanisms, including transformation of naturally competent bacteria, phages, and the use of conjugative plasmids, transposons, and integrons. Despite barriers to the exchange of genetic information between different genera of bacteria, widespread transfer of resistance genes from chromosomes of environmental and soil bacteria to the mobilizable elements in clinical isolates seems to have occurred. Indeed several examples of recent transfers from environmental bacteria to the clinical strains are available (Route 3, Figure 2); however, very limited evidence for recent direct transfer from producers to clinical strains has been obtained (Route 1, Figure 2). Nevertheless, transfer from producer bacteria to other actinomycetes in soil is possible (Route 2A), which could provide a pathway for further transfer of these determinants to proteobacterial clinical strains (Route 2B). Based on the available evidence, we conclude that Routes 2 and 3 are much more prevalent in nature as compared to Route 1 for transfer of resistance genes to pathogens.

To better understand factors that promote dissemination of resistance genes and to elucidate relationships between antibiotic resistance genes of producer, environmental, and pathogenic bacteria, new and improved strategies for sampling and screening of microbial populations and metagenomic libraries are needed. Moreover, better algorithms and the use of bioinformatics approaches for determining relationships between resistance determinants of different environmental niches will be highly beneficial. Additional genome sequencing data will also help fill the gaps in our knowledge of intermediate stages and carriers for mobilization. Indeed two databases, the Antibiotic Resistance Database (ARDB) and the Comprehensive Antibiotic Resistance Database (CARD), assembled in the last decade (Liu and Pop, 2009; McArthur et al., 2013), are expected to provide computational tools for the rapid prediction of antibiotic resistance genes and their targets in newly sequenced genomes and establish phylogenetic relationships. This was demonstrated in a recent bioinformatics study using these databases (Jiang et al., 2017). It is expected that these bioinformatics tools will unify information on resistance genes and their products found in thousands of bacterial species isolated from the clinic or the environment as well as their associated mobile genetic elements and allow this information to be quickly mined by researchers in this field.

## Author Contributions

PK supervised the work, collected and reviewed literature, and co-wrote the review article. EP collected and reviewed literature, prepared the figures/tables, and co-wrote the review.

## Conflict of Interest Statement

The authors declare that the research was conducted in the absence of any commercial or financial relationships that could be construed as a potential conflict of interest.
